# From theory to practice: a novel meditation program at a global corporation

**DOI:** 10.1007/s12144-023-04516-1

**Published:** 2023-04-19

**Authors:** Anne V. Weisbrod, Lisa C. Bohman, Krystyn J. Ramdial

**Affiliations:** 1 Resilience Arts & Science, LLC, Cincinnati, OH USA; 2 Global Sustainability, The Procter & Gamble Company, 8700 S. Mason Montgomery Road, Cincinnati, OH 45050 USA; 3 Advanced Consumer Modeling & Statistics, The Procter & Gamble Company, Cincinnati, OH USA; 4 Global Talent Development, The Procter & Gamble Company, Cincinnati, OH USA

**Keywords:** Virtual intervention, Stress resilience, Empathy, Meditation, Mindfulness, Health coaching

## Abstract

Global levels of stress, worry, sadness, and anger hit new highs in recent years, and employee well-being has been identified as a necessary focus in occupational health. Developed over 6 years in a large multi-national company, the Meditation Without Expectations™ 8-week course evolved from theories to practice. The intervention teaches 8 meditation techniques in a specific order and incorporates health coaching and adult learning principles that drive impact. The wellbeing program was offered using a virtual online platform to employees in more than 30 countries during 2021-22. Its effectiveness was evaluated using established standard questions and cutting-edge consumer research methods. The descriptive study uses quantitative and qualitative analyses from more than a thousand employees. Paired t-tests are used to compare pre- and post-course survey scores. The test subjects who completed the 8-week course had significant improvements (p < 0.0001) across genders, geographies, and durations of employment, and in all measured domains of stress, mindfulness, resiliency, and empathy, whereas the comparison group did not. Advanced topics analysis is used to extract common learning objectives from unstructured text submitted by enrolled employees, which helped focus the intervention on what people need or want to learn. A proprietary artificial intelligence model is used to classify subjects’ comments after completing the course, finding highly positive outcomes with potential for new habit creation due to a mental model change. A framework of characteristics that make the intervention impactful is also shared.

## Introduction

The Society for Industrial and Organizational Psychology’s Top 10 Work Trends for 2022 lists three topics related to occupational health: (1) an employer’s role in employees’ mental health, (2) stress and burnout, and (3) caring for employee wellbeing (SIOP, 2022). While this is the seventh consecutive year that employee health and wellbeing has been identified as a necessary focus, this is the first time that it has been broken down into three distinct topics, emphasizing the continuously growing need for intentional research and effective intervention in this area.

Although the positive and negative emotional experiences of people globally vary widely, a 2018 survey by Gallop of 151,000 people in 140 countries found global levels of stress, worry, sadness, and anger hit new highs. More than 50% of interviewees in some countries, like the US, reported high stress and other negative emotional experiences (Gallop, 2019). A meta-analysis of 30 studies from a dozen countries found stress, anxiety, depression, and insomnia increased significantly during the COVID-19 pandemic relative to 2019, and subsequently declined (Mahmud et al., 2022). That said, the 2022 Pandemic Anniversary Survey conducted by The Harris Poll on behalf of the American Psychological Association reveals that concerns about inflation and war have pushed stress to unprecedented levels that challenge Americans’ ability to cope (APA, 2022).

Such emotional strain, in combination with workplace pressures, can have major consequences at the organization-level, strengthening the business case for organizations to invest in employee health and wellbeing. Not only can unhealthy employees directly impact the financial bottom line of organizations, but they can also contribute to indirect costs driven by productivity loss, workplace injuries, performance issues, attrition, and absenteeism (Fogarty, 2008; Harter et al., 2002). Of course, leaders of ethically responsible organizations also do not want colleagues to feel dissatisfied or miserable at work. The significant impact of COVID-19 conditions on health and well-being, and its repercussions on already high work demands, is what led to the development of the meditation-based program in this paper.

One of the most studied organizational intervention approaches to encourage occupational health improvements in these areas is Mindfulness-Based Programs (MBPs), which include a variety of theoretical and therapeutic ways to train adults in mindfulness practices systematically (Crane et al., 2017). Stress reduction and management, and increased focus, are two commonly desired outcomes of such programs.

While the extant literature supports the positive impacts of MBPs, there is much more to learn to enable organizations in their efforts to justify the investment and deliver effective meditation-based programs. The theoretical rationale of why mindfulness training might positively impact work outcomes is logically defensible, but the published evidence appears to largely come from health and education sectors, generally small samples with some quality unknowns or limitations, and simple quantitative surveys with standard questions. Very few studies evaluate the impact of MBPs across multinational employee populations, as online interventions, or how participants really feel about the intervention. Thus, other sectors and global organizations have started, but have been slow to invest in, wide-scale delivery of MBPs in the workplace.

A meta-analysis of workplace mindfulness studies cataloged 25 separate studies, of which 15 studies included data from less than 90 people, 8 studies included data from between 100 and 200 people, and only 2 studies included data from more than 200 people (Bartlett et al., 2019). Of those 25 studies, 10 were of people working in health settings (e.g., nurses and hospital-based health care staff) and another 9 studies included samples of those working in education (e.g., schoolteachers and university staff). Furthermore, only two of those studies used an intervention that could be delivered online.

Eby and colleagues (2019) provide another comprehensive review of 67 published studies, highlighting a variety of design, delivery, and evaluation methods for MBPs. 80% of these programs were focused on stress reduction. Most were evaluated using a pre-course and post-course survey using standard questions and did not employ an active control or check of internal validity. Most studies (55) had samples between 3 and 98 and were conducted for the health (33) and education (16) sectors. Our review of the literature reveals almost no studies of MBPs with participants from multinational organizations.

While the investigative and statistical approaches used in MBP research to date have encouraged trial and adoption, this study uniquely leverages advances in consumer products research to provide additional valuable insights to deepen our understanding of employee mental health, stress, and wellbeing. Organizations that provide goods and services often conduct research on consumers to understand their motivations and product use behaviors. We hypothesized that consumer research methods can be used to better understand both the motivations behind employee participation in wellbeing interventions, and the mechanisms behind behavior changes associated with those interventions. With the purpose to advance understanding of what is effective for mental and emotional wellbeing interventions, especially in multi-cultural global organizations, the objectives of this paper are to: *(1) share findings from a large international sample participating in a new virtual meditation-based program; (2) reapply established MBP standard questions and novel statistical methods used in advanced consumer products research to evaluate effectiveness of the new meditation-based program on employee stress, mindfulness, empathy, and resilience; and (3) propose a framework of characteristics that make a mental and emotional wellbeing intervention effective.* We intend for the methods and results to be useful for people studying, purchasing, or developing an effective MBP for their large organizations.

## Theoretical background

### Meditation and mindfulness

The general term Meditation refers to a wide range of practices, such as techniques to promote relaxation, exercises to increase physical or emotional awareness, or contemplation of a situation while being silent and still; practices may involve secular or religious approaches to focus on a specific object or subject, or open effortless monitoring of the moment (Lutz et al., 2008).

There are two meditation styles that we will define for the purpose of this paper: mindfulness, or focused attention, and open monitoring.

Mindfulness is a focused-attention or concentrative meditation in which a person focuses nonjudgmental awareness on one aspect in the present moment. In this approach, the mind focuses on an object, the mind wanders from the object of focus, there is awareness of the mind wandering, there is conscious effort that shifts attention back to the object of focus, and then there is sustained attention (Hasenkamp & Barsalou, 2012). The object of focus could be external or internal, such as watching a candle burn, or repeating a short phrase/mantra or counting. Through such practices that encourage concentration, participants learn to actively redirect attention to the object of focus, which eventually improves attention span (Petranker & Eastwood, 2021). Focused attention practices that increase awareness of emotions, such as loving-kindness or “metta” as it is called in the Pali language and other compassion meditations, can improve aspects of emotional intelligence (EI) and social connections, which are pivotal in lifestyle medicine and productive working relationships (Hofman et al., 2011; Yang et al., 2016). EI involves the ability to be aware of, regulate, and express emotions and to understand, and respond appropriately, to the emotions of others. In this corporate context, EI skills are associated with affective communication that build positive working relationships and teams, and collaborative innovation to solve challenges.

Other kinds of meditation approaches fall under the term open monitoring or non-directive meditation, in which attention is open to all experiences, the many details and interconnections occurring. Meditators may witness “everything”, such as listening to all sounds in the present moment, while feeling the body breathing, subtle emotions and physical tensions, and noticing inner speech without focusing on it. Such techniques are described as ‘effortless’, ‘not doing’, or ‘just being’, which once learned, are associated with high-level cognitive and better executive function (Tanaka et al., 2022).

Dubbed the ‘Mindfulness Revolution’, the increasing trend is to train employees in meditation and contemplation practices, sometimes in concert with physical exercises or more typical business leadership development programs. An extensive landscape study by Ihl et al. (2020) demonstrates that the success, or lack thereof, of mindfulness programs depends on their content and execution. The authors found that well-founded MBPs, provided through systemic interventions, can produce positive outcomes such as stress reduction and increased resiliency. Also, some programs do not deliver the desired results because they are poorly informed and/or have unreflective implementations that miss the point of mental or emotional health and development. The ineffective interventions are inappropriately lumped under the popular term “mindfulness” and driven more by business acceleration goals than individual wellbeing or community development.

## Adult learning and behavior change

According to a recent review of more than 200 studies, health and wellness coaching appears to be a promising intervention strategy for driving lifestyle behavior changes (Sforzo et al., 2017). Health coaching can help people to understand and simplify the complex process of changing his/her/their entrenched behaviors, by communicating the importance of individual awareness, choice, and execution. The health coach credentialling manual by Muth (2019) notes that after a client realizes that a behavior change could be helpful, then he/she/they will actively choose which new actions to take, based on their strengths and other support and circumstances. Feeling accountable and evaluating progress through specific actions also enables a person to achieve and maintain the desired change. The program in this study uses common coaching and meditation practices to direct people in a virtual large group setting to become aware of physical, emotional, and mental sensations, to explore their motivations and new effective strategies based on meditation techniques, and thus change how they view and respond to potentially stressful situations.

There are multiple theories and successful approaches to adult learning. The meditation-based program in this study incorporates six core principles of adult learning in the session format, as described by Knowles and colleagues (2020). First, adults seek out and retain information when they actually need it (1). There is a problem they want to solve (2), so they become oriented toward learning the new information and skill (3). When they are ready and feel intrinsically motivated to learn (4), they are more likely to retain information and make changes in behaviors. Feeling autonomous and directing their own exploration of new information and habits (5), benefits learning. Finally, new information and practices that build on prior experiences (6) are also more likely to be understood and retained.

There are many effective models that describe stages of behavior change; the transtheoretical model (TTM) of behavior change is a popular and practical approach increasingly used by health coaches (Prochaska & Velicer, 1997). TTM posits that a person moves through six steps: precontemplation, contemplation, preparation, action, maintenance, and termination. In theory, an effective organizational wellbeing program would offer several options for people in each stage of behavior change related to a wellbeing topic, thus aiding their movement toward action and maintenance of a positive health change. Short lectures or workshops, and repeated information such as through an app, can introduce people in the precontemplation and contemplation stages to concepts and basic wellbeing practices. Longer programs can support people to prepare and to take specific actions over weeks or months. The program in this study is intended for participants in the TTM preparation and action stages for improving mental and emotional health and wellbeing. At the company hosting the program in this study, different but related meditation programs run every week throughout the year, supporting people in the TTM maintenance stage.

Building rapport within an online group of colleagues who have not met before takes concerted effort, which can be aided by virtual coaching techniques such as the CRAFT model (Ross, 2019). CRAFT is an acronym. Sessions begin with a Check-in by the coach and client(s), in which they informally share personal experiences from the week to help to develop familiarity and relationship. That is followed by an open discussion of the coaching topic, in which the client(s) Reports on their progress and ask questions. The ensuing conversations enable clients to Adjust their skills to practice or alter or choose Assignments for the next week’s homework. Feedback is collected in-the-moment and the coach provides Teaching/Training throughout the session. The new meditation-based program in this study incorporates the CRAFT model in its format.

### Interventions for behavioral change

Wellness interventions designed to support learning and behavioral change tend to focus on the undesirable cognitions or emotions, or on coping with the problem or stressor that is leading to the strain on wellness (Lazarus & Folkman, 1984). While both have their merits, we will focus on the cognitive and emotional behavioral changes as they are most relevant to this study. Cognitive-behavioral intervention methodology branches from cognitive-behavioral therapy which focuses on the acknowledgement and acceptance of thoughts and emotions with the intent of enabling a person to adjust their perceptions of those thoughts and emotions. Rather than seeing unpleasant cognitions and emotions as threatening, people are encouraged to experience them without trying to avoid or control them. This methodology has been found to improve mental health outcomes and work-related variables (Bond & Bunce, 2000).

While we may not always consider these types of interventions as a part of our work environment, there has been research to demonstrate the impact that they can have when they are integrated into our work lives. A meta-analysis conducted on MBPs delivered in the workplace, when executed well, can have long-lasting effects of at least 12 months for stress reduction and overall mental health and well-being (Bartlett et al., 2019).

#### Meditation without expectations^TM^ (MWE) – this study’s intervention

Developed over six years through trainings with thousands of employees for a large multi-national corporation, the MWE 8-week virtual course teaches eight meditation techniques in a specific order, incorporates health coaching, adult learning, and behavior change principles. The initial goal was to enable participants to develop their self-sufficiency in implementing a consistent meditation practice. The program was first offered via livestream in English across all time zones starting in the first year of the COVID-19 pandemic, as many employees switched to working from home.

The program was developed and led by a very experienced meditator, health coach and scientist at the corporation, in response to common needs and interests expressed through direct requests and internal employee surveys. Aligned with the adult learning principle that adults are more likely to participate in programs that address their specific needs, this program was designed to directly address problems the employees identified. Written anonymous feedback was collected from over 1,500 program enrollees around the world, which highlighted that their top needs for a corporate intervention were to: (1) Reduce anxiety, distraction, negativity about self and world; (2) Increase adoption of essential mental and emotional wellbeing skills; (3) Improve quality of life and work performance (e.g. increased focus, patience); (4) Be reassured of participation by having a company-supported program offered during the workday; (5) Feel understood and trust a competent credible teacher in their corporate culture; and (6) Be part of a regular practice community for encouragement, accountability, insight, and friendship.

The meditation techniques taught through MWE are secular, including: Being Here with Awareness through body scan and 14 + senses (Bradford & Harvey, 2022); Introspection and Gratitude: reward-based learning/R.A.I.N. (Brewer, 2017) and Naikan therapy (Krech, 2001); Dealing with Distractions: counting and mantra; Compassion: metta and tonglen (Gheyzen & Delameilleure, 2020); and Choiceless Awareness: open monitoring/not doing.

The CRAFT Model from group and individual health coaching is incorporated into the structure of each week’s session (Ross, 2019). Additionally, techniques on how to choose and take small steps to change habits (Fogg, 2020), cognitive behavioral coaching (Green et al., 2006), and facilitating group learning (Knowles et al., 2020) are also incorporated into the MWE format.

MWE is designed to be highly experiential. Each week, the 50-minute session includes a check-in and progress report by participants, a 15-minute lecture on background and approach to the meditation for the week, 15- to 20-minute meditation practice (guided or silent), and discussion of their experiences and questions via online chat and voice/video.

This is novel versus common MBPs due to the structure of the MWE program, the instructor, and the participants. The MWE program was developed to be effective across cultures, offered solely through a virtual platform, and integrates health coaching, adult learning, behavior change, and meditation techniques and perspectives that are not part of the construct of other MBPs. The instructor is a highly experienced veteran relative to most ‘wellbeing’ teachers marketing their services to corporations, with more than three decades of meditation practice, education, and certifications in several related fields. In our literature searches of hundreds of articles on meditation studies, none cover a global corporate population working in many countries and only a dozen studies have sample sizes larger than 90 subjects (vs. this study of 268–1079 people in > 30 countries).

### Characteristics of effective MBPs

In addition to best practices in adult learning, there are factors that contribute to an intentionally well-designed and structured wellness intervention. A comparative analysis of wellness programs across Europe identified a five-phase model of elements which seem to emerge across effective programs (Nielsen et al., 2010). These five phases include: (1) preparatory activities, such as determining the drivers of change; (2) screening of current systems and selection of intervention methods; (3) action planning to design the intervention; (4) implementation of the intervention; and (5) evaluation of the intervention. The five phases continue in an ongoing loop, with a critical element surrounding them – employee participation. Employee participation is believed to be critical for several reasons, notably that it empowers employees to take ownership over their learning experience.

As we consider MBPs more specifically, Crane and colleagues (2017), make several recommendations for designing an effective program. The program should have a theoretical foundation based in medicine, psychology, and education. It should be intentional about addressing and relieving the causes of human distress, enable the participants to develop a healthy relationship with their experience of the present moment, support improvements in self-regulation (e.g., emotions and behaviors) and compassion, and employ a sustained experiential inquiry-based learning process. Aside from the program content, the characteristics of the instructors also carry importance. Instructors of effective programs should have the right skillset and competencies, be capable of role modeling the behaviors and qualities they are teaching, engage in ongoing training and practice, and continue to be a part of the participatory learning process along with the participants.

The Kirkpatrick Model for evaluating trainings recommends a comprehensive evaluation plan with 4 levels of assessment: Reaction, Learning, Behavior, Results (Kirkpatrick & Kirkpatrick, 2016). Level 1 is an evaluation of each participant’s reaction to the program: how much the person enjoyed it, and degree of engagement and relevance. Level 2 is an evaluation of each person’s knowledge, skills, attitudes, confidence, commitment resulting from the experience, which usually includes a pre- and post-program evaluation test to determine what and/or how much was learned. Level 3 measures behavioral change resulting from training/coaching experience. Level 4 measures hard results for the return on investment, such as progress toward or impact on a person’s health, family, work performance, or community.

## Outcomes of MBPs

Well-executed MBPs have the potential to equip employees with skills that enable them to navigate work-induced stress. One well-known theory that helps to illustrate this is the Job-Demand-Control-Support (JDCS) model (Karasek & Theorell, 1990). The JDCS model posits that, while job demands can lead to employee stress, this stress can be buffered by job skills that enable employees to increase work-related support and control. Although the originally proposed definition of control within this theory has centered around control of the work through job characteristics such as autonomy and decision-making authority, research has found evidence that perceived control is enough to elicit a decrease in stress and strain (Kushnir & Melamed, 1991). In addition to perceived control, the role of perceived social support in the workplace is also important for mitigating the negative impact of job demands. The most impactful forms of support are expected to come from those within employees’ job-related network, typically the managers and coworkers. Considering the role of control and social support in supporting employee well-being, it is reasonable to expect that an intervention designed to increase perceived control and support will have positive outcomes related to employee wellbeing.

It has been posited that attention is a key mechanism in how mindfulness impacts several outcomes, such as attentional stability, control, and efficiency (Good et al., 2016). Attention is also an important component in developing awareness and control over emotional, cognitive, and physiological processes (Jamieson & Tuckey, 2017). Mindfulness techniques focused on attention have also been found to create High Reliability Organizations, efficient entities with very low failure rates, such as healthcare where failure carries a very high cost (Nales & Chakravorty, 2016). In their paper, Nales and Chakravorty connect mindfulness techniques such as quiet meditation, mindful reflection, and communication to an employee’s ability to stay alert and focused on tasks and to understand how actions influence the people they serve. Those behaviors, in turn, would then enable the healthcare organization to increase the release and survival of patients. For a corporate manufacturing setting, staying alert and focused might be extrapolated to imply efficient production or fewer safety incidents.

## Consumer research approach

Consumer research spans a wide range of methods, input data, and objectives. Its results provide information for product development and communication strategies and contribute to business decisions. Methods can measure how a consumer uses a product, which aspects of product use impact their appraisal of a product, which new product attributes will impact purchase, what drives repurchase, and more (IDEO.org, 2015). One commonly used method is to collect data before and after a consumer uses a product, measuring how a consumer’s experience changes with product use. Standard survey questions with close-ended questions are frequently used to develop quantitative measures. Although helpful, researchers can miss some important vectors, like emotion, about the consumer experience when only such questions and scales are used. Qualitative responses from open-ended questions can add insight into how the product experience impacts the user. This unstructured text can provide a unique view from what consumers say in their own words, delivering clear context, and referencing what is most important for them. The quantitative and qualitative consumer research methods applied in this study were derived from the best practices of a company known worldwide for its extensive consumer research and development of product testing methods; these methods are applied in a new way to gauge whether the 8-week MWE program could impact subjects’ stress, resilience, mindfulness, and empathy.

To collect the qualitative data, this study leverages Natural Language Processing (NLP) which identifies increasing emotional engagement levels driven by a product experience, also known as a Mental Model Change (MMC). Consumer product developers at the company recognized that not all top 5-star ratings were equal, so they collaborated with an expert vendor to develop an artificial intelligence NLP-based model to gain more insight and classify consumer comments into 6 levels of reaction to a new product: Negative, Neutral, Good, Great, WOW, and MMC. A Negative score signals that the consumer dislikes the product; such comments can be helpful to learn what needs to be fixed. A Neutral score means the product has low value to the consumer. A Good score designates that the consumer has positive feelings but is uncertain about buying the product in future. A Great score indicates high enthusiasm and positive feelings about the product. A WOW score specifies the experience is very special and evokes surprise, love, and delight in the consumer, as in ‘the product wow’ed them’. A MMC score indicates a paradigm shift: what the consumer thought was the best product they have ever used, suddenly changes, as they use the new product that redirects how they think, feel, and act as a result of the experience. This is demonstrated with comments like ‘I never thought that XYZ product could do ABC, but this product really does!‘ The experience has changed their mental model of the product category. A high percentage of WOW and MMC scores are indicative of a novel outstanding experience.

## Methodology

### Participants and procedure

MWE is a live-streamed course offered globally to employees of the company. The course is marketed internally through its daily news service and word of mouth, usually by previous participants. Participation is self-selected, voluntary, and free for any employee. It is marketed as appropriate for people with no, a little, or a fair bit of experience with meditation or other mind/heart training practices. Participants are strongly encouraged to attend all classes (50 min each week for 8 weeks), and to practice on their own. Weekly participation and course completion is not officially tracked but is reported by survey respondents. Employees are dominantly college educated, with a range of familiarity with the English language from basic to proficient. See Table 1 for participant demographics.

**Table 1:** *Demographics, as reported by course participants voluntarily answering the pre- and post-course survey.* *268 subjects completed both pre and post surveys for their session.*

Demographic Variable	Count (Percent)
**Gender**	
Female	198 (73.9%)
Male	65 (24.3%)
Prefer Not to Answer	4 (1.5%)
Transgender	1 (0.4%)
**Years of Employment at company**	
Under 10	81 (30.2%)
10–20	60 (22.4%)
20+	127 (47.4%)
**Number of Classes Attended**	
4 or fewer	34 (12.7%)
5	34 (12.7%)
6	63 (23.6%)
7	66 (24.7%)
8	70 (26.2%)
**Region of Employment**	
United States	123 (45.9%)
Europe	95 (35.4%)
Canada	22 (8.2%)
Latin America	11 (4.1%)
Southeast Asia	9 (3.4%)
China	4 (1.5%)
India	2 (0.7%)
Middle East / Africa	2 (0.7%)
**Meditating Prior to Course**	
No	131 (48.9%)
Yes	137 (51.1%)

Over 1,000 participants who attended the pilot programs held January through May 2021 were asked upon registering to indicate their “objectives for attending and completing this course.” This serves two central purposes: (1) when participants consciously recognize their unique learning objective(s) or motivation(s) to attend a course, they are more likely to commit their time and participate fully, and (2) the responses yield insight into the mental and emotional aspects (e.g., anxiety and distraction) that the program must target to be an effective intervention.

Participants enrolled in the program January 2021 through June 2022 were asked to complete a survey containing 50 standardized questions, commonly used to evaluate MBPs. Participation in the survey was voluntary. Participants used a unique identifier code to ensure their responses were anonymous. They self-reported gender, years of employment at the company, region of employment, and whether they had a current meditation practice. It is important to note that region of employment is just roughly related to nationality, as the nature of having international operations means people are moved to work in other countries regularly.

The pre-course survey was sent 1 week prior to the beginning of the course and the identical post-course survey was sent after the last class in week 8. Participants received email reminders three times for each survey. Paired pre- and post- survey responses from the courses offered in September – November 2021, January – March 2022, and May – June 2022 are used for this statistical analysis (*N* = 268). The test group includes people who attended 5 or more classes in an 8-week session (N = 234). The comparison group (N = 34) includes people who initially enrolled in the program but dropped out. These subjects attended between 1 and 4 classes. Through direct survey comments, and our interpretation of the qualitative open-ended comments, there were several reasons for dropping out: (a) stage of readiness: people are in the TTM model stage of contemplation or preparation and thus not yet ready to take action, i.e., actually call in. This might also be explained as not prioritizing class attendance over other work demands; (b) unsupportive workplace: employees felt discouraged from taking time at work to learn such new skills or improve their health; (c) employment leave: some subjects left work temporarily e.g., medical leave; (d) not their thing: some people did not like the course format or practicing meditation. Also, some participants shared that there is a stigma in their culture against practicing meditation where it may be perceived as a cult practice or a weakness that one needs such a coping strategy.

### Measures

Participants answered the standardized questions pre- and post- session, which captured four latent domains: stress, mindfulness, empathy, and resilience. This longitudinal data collection allows the test of changes taking place over the 8-week session. Each domain is assessed using previously validated questionnaires, as described below.

**Stress** is the physiological and/or psychological response to external stimuli perceived as a threat or unexpected negative change. The Perceived Stress Scale (PSS) is the most widely used psychological instrument to assess the stressfulness of situations and the effectiveness of stress-reducing interventions (Cohen et al., 1983). The 10 standard statements in PSS tap into how unpredictable, uncontrollable, and overloaded respondents find their lives, and their perceived level of helplessness and self-efficacy. For example, an item asks participants to indicate how frequently they *“Found that you could not cope with all the things that you had to do?”* on a 5-point scale. Two subscales are surveyed: (1) **Overloaded** – capacity filled to excess so that function is impaired; and (2) **Uncontrollable** – incapable of being managed or corrected, implying turbulence, disorder, or threat.

**Mindfulness** is assessed using the Five Facet Mindfulness Questionnaire (FFMQ), which investigates a person’s ability to: Clarify goals, Accept negative thoughts but not react to them, Cultivate openness and awareness of mind and body, Deal with stress and adversities, Express and regulate emotions, Solve problems and make decisions, and Nurture positive relationships (Baer et al., 2006). For example, participants were asked to indicate the extent to which statements like *“I don’t pay attention to what I’m doing because I’m daydreaming, worrying, or otherwise distracted.”* on a 5-point scale. There are 15 statements across 5 subscales in the FFMQ: (1) **Observation** - the ways we see, feel, and perceive the internal and external world around us and select the stimuli that require our attention and focus, (2) **Description** - how we label our experiences and express them in words to ourselves and others, (3) **Acting with awareness** - whether we can act with judgment and get out of the autopilot mode before responding to a situation, (4) **Non-judgment** of inner experience - whether we let the inner critic take a toll on our happiness and positive state of mind, and (5) **Non-reactivity** to inner experience - active detachment from negative thoughts and emotions so that we can accept their existence and choose not to react to them to make way for emotional resilience and restore mental balance.

**Empathy** is the capacity of an individual to understand, feel, and react to the observed experiences of another person; the capacity to place oneself in another’s position and to respond with an appropriate (productive) emotion. Empathy is assessed using the Interpersonal Reactivity Index (IRI); this standard measurement tool has been cited over 10,000 times (David, 1983). There are two subscales in the IRI, with 7 questions in each: (1) **Perspective Taking** - the tendency to spontaneously adopt another persons’ psychological point of view, and (2) **Empathetic Concern** - “other-oriented” feelings of sympathy, concern, and compassion. For example, participants are asked to indicate how frequently they *“…try to understand my friends better by imagining how things look from their perspective.”* on a 5-point scale.

Although the originally published IRI standard questions were used, answer options were modified following complaints from non-native English speakers that some of the statements and answer options were confusing. The original IRI answer options are: Does not describe me well, Describes me a little, Neutral, Describes me somewhat well, Describes me very well. The answer options were changed in this study to: Never, Almost never, Sometimes, Fairly often, Very often. This retains the 5-point scale and reapplies the Likert scale known to work in the PSS.

**Resilience** is the ability to bounce back or recover from stress: adaptation, thriving, and resistance to illness, physical or mental strain. Resilience is thought to buffer the impact of high emotional demands and maintain engagement when job demands are high. The brief resilience scale (BRS) is a 6-question self-report tool that predictably relates personal characteristics, social relations, coping, and health (Smith, 2008). For example, participants are asked to indicate the extent to which they agree with statements such as *“It does not take me long to recover from a stressful event.”* on a 5-point scale.

### Analyses

The unstructured text from the pre-course question asking for subject objectives were analyzed in two ways: Word clouds and Topic analysis. The text collected (*N* = 1,079 responses) had an average length of 14 terms. Text cleaning was performed prior to analysis; terms were at least 4 characters in length, some words were classified as stop words to skip over (e.g., articles like ‘the’), and some phrases were created from the statements (e.g., ‘mental health’). Words or phrases had to occur at least 15 times to remain in the analysis. There were 912 unique words, and 6342 total words. Word clouds were based on the frequency of terms and phrases. Singular value decomposition (SVD) was used to identify underlying Topics in the text. SVD is a way of exploring how words are used together in subject responses. Comment Topics identified in these data were based on SVD and wellbeing expert advice on common terms and relationships among the terms. These analyses were performed in JMP® Pro Version 16.0.0, SAS Institute Inc., Cary, NC, 1989–2021. Consumer research experts also read all the verbatim comments and tabulated trends by hand to gain insight and confirm whether the software produced understandable Topic groupings.

The unstructured text from the post-course feedback (*N* = 471 responses) were analyzed as described above and using a special model evaluating a MMC. This is an artificial intelligence (AI) program that was developed over a decade from tens of thousands of consumer comments and ratings of products. The MMC model leverages words that express positive emotion and delight, superlatives, relative comparisons against other products, or indicate a resetting of the user’s reference for what a product is or does. This MMC model is not a generic sentiment analysis package; billions of dollars of product investments depend on the reliable results of this proprietary AI program. Machine learning is used to classify text, and as consumer research data are added to the database, the algorithm relearns and improves. As part of this process, researchers also send direct feedback to the programmers identifying specific comments that are misclassified so that the algorithm may be further corrected and optimized. The MMC algorithm was run in Python® and JMP® was configured to integrate with that analysis.

Responses to the PSS, FFMQ, IRI, and BRS questions in the pre- and post- program surveys were combined into their respective domain. Changes in domain means were calculated by subtracting the pre-session domain means from the post-session domain means. Data were first analyzed with a linear mixed model with a random subject effect and fixed session effect to evaluate session to session variability (O’Connell et al., 2017). As session was not a significant effect (*P*-values > 0.30), it was removed as a variable from the analysis. Paired t-tests are used to compare pre- and post-session means of the data combined from all three sessions. All testing was 2-sided, and the level of significance was alpha = 0.05. Domain reliability is evaluated with Cronbach alpha, Pearson correlations, and Cohen’s d statistics. Analyses were performed in SAS® 9.4 software, SAS Institute Inc., Cary, NC, USA. Analysis was also conducted on participant subgroups to assess whether subpopulations experienced unique improvement. Subgroups include gender, number of years of employment with the company, region of employment, and the number of classes attended.

## Results

### Employee learning objectives

**Fig. 1:**
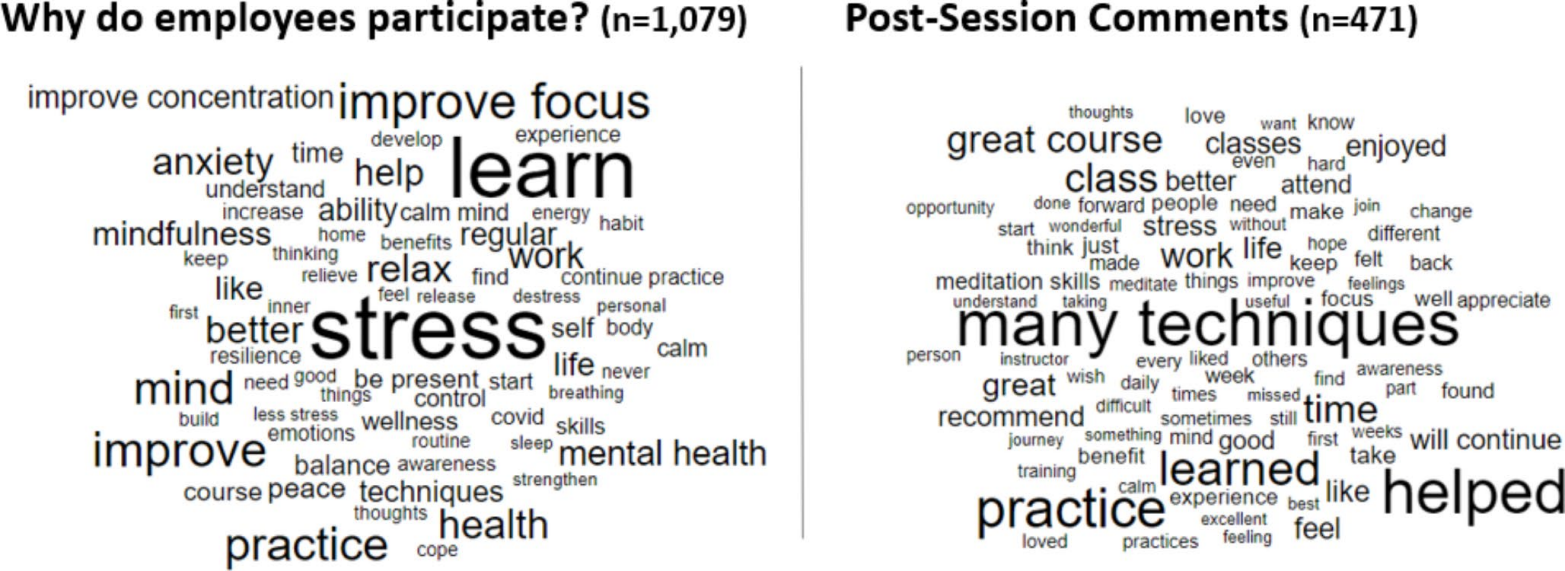
*Word clouds from responses to open-ended questions in session surveys that depict employee pre-session learning objectives (N = 1,079) and post-session comments (N = 471) describing what participants learned and felt*

Figure 1 shows the word clouds incorporating all subjects’ pre-course objectives for enrolling in the program, and post-course comments about what participants learned and felt, helping to evaluate whether employees’ original learning needs were met.

The first word cloud gives visual insight into why employees wanted to participate, such as reducing stress, learning, improving focus/concentration and mental health. Using that same text expressing their pre-course objectives, Topic analysis identified that there are 5 groupings or topics that represent common objectives for people enrolling in the course:

1. to improve mental health and reduce stress – top loadings (0.80 − 0.27) include the terms: mental health, health, life, wellness, and stress (negative loading, -0.20).
2. to feel more at peace and calm – top loadings (0.66 − 0.43) include: mind, body, peace, and stress (negative loading − 0.30).
3. to improve emotional intelligence and feel life is manageable – top loadings (0.48 − 0.34) include: emotions, find, control, calm, balance.
4. to increase focus/concentration - top loadings (0.49 − 0.29) include: improve focus, improve concentration, ability, work, better, help.
5. to learn and practice – top loadings (0.49 − 0.34) include: practice, like, course, understand, learn, regular.

These Topics appear consistent with the themes revealed through the word cloud. Word clouds are visually appealing and useful as infographics to communicate general themes, however topic analysis with loadings provide additional clarity on how the words are used by analyzing the key word groupings into topics. Text cleaning and understanding relationships between terms is important to produce understandable groups with either method.

### Pre-post survey data quality

**Fig. 2:**
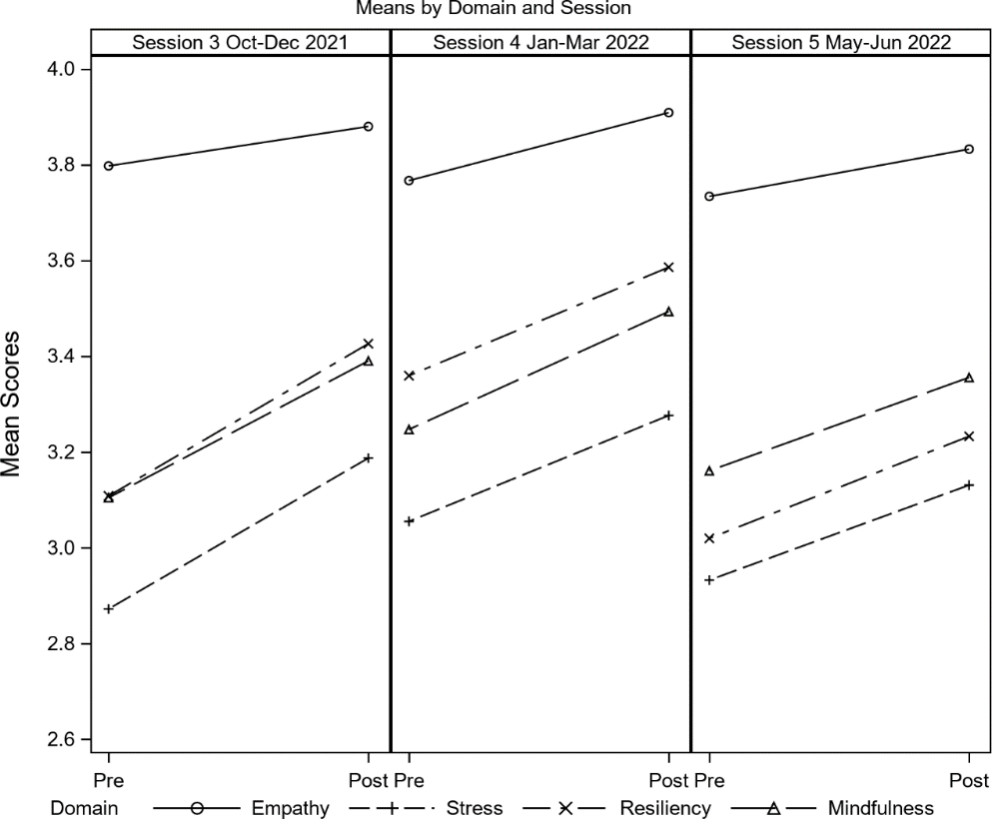
*Pre and post program domain means for each MWE session in this study (N = 268)*

Evaluation of the paired pre- and post-session survey data from the 3 sessions with a linear mixed model showed that there is no significant impact of time (i.e., session) on the subject responses, that the data follow a normal distribution, and the domain results are reliable. Figure 2 shows domain means by session, providing a visualization that panelists showed improvement in all domains for all sessions. Statistical testing using paired t-tests confirmed that these improvements were statistically significant.

Survey measures were evaluated using Cronbach alpha, Pearson correlation, and Cohen’s d for the four domains (Table 2). All domains showed consistency of measurement as indicated by Cronbach alphas, which are greater than 0.75, representing a range of acceptable to good internal consistency. Impact of the program is measured by Cohen’s d, which show a range of impact from low to medium size. Values for Stress and Mindfulness are 0.46–0.50, which is generally interpreted as a medium-sized effect. Empathy and Resiliency domains showed low to medium effects (0.25–0.35). Pearson correlations show a good test-retest correlation of roughly 0.7. Histograms (not shown) of mean changes by MWE participants from all sessions and domains demonstrate a normal distribution with symmetry and a classic bell shape; the centers of the distributions are to the right of zero, aligning with the overall improvements presented in Table 2.

**Table 2:** *MWE pre- and post-session means, standard errors, mean differences, t statistics and p-values by domain for two panelist groups, with reliability measures (Cronbach alpha, Pearson correlation, Cohen’s d)*

*Group/Domain*	*Pre Course Mean (StdErr)*	*Post Course Mean (StdErr)*	*Mean Difference* ^*a*^	*Std Err Difference*	*t Statistic*	*P-value* ^*b*^	*Cronbach Alpha*	*Pearson Correlation*	*Cohen’s d*
**Test Group (*N*** **= 234)**									
Empathy	3.77 (0.426)	3.88 (0.432)	0.12	0.022	5.44	< 0.0001	0.84	0.72	0.25
Stress	2.95 (0.551)	3.20 (0.554)	0.28	0.028	9.81	< 0.0001	0.81	0.69	0.46
Resiliency	3.17 (0.778)	3.43 (0.694)	0.29	0.036	7.93	< 0.0001	0.84	0.72	0.35
Mindfulness	3.17 (0.505)	3.42 (0.492)	0.26	0.028	9.18	< 0.0001	0.78	0.64	0.50
**Comparison Group (*N*** **= 34)**									
Empathy	3.84 (0.082)	3.87 (0.074)	0.03	0.042	0.70	0.4888			
Stress	2.86 (0.092)	2.96 (0.104)	0.10	0.079	1.26	0.2150			
Resiliency	3.02 (0.123)	3.10 (0.130)	0.07	0.087	0.85	0.4015			
Mindfulness	3.22 (0.093)	3.40 (0.092)	0.18	0.060	2.97	0.0055			

^a^ *Question scales were 1 to 5. Positive mean difference indicates improvement.*

^*b*^ *Pre- and Post- session data were analyzed with a paired t-test by domain.*

### Pre-post program results

All four domains showed significant improvement when comparing the pre- and post- program survey responses. Table 2 lists domain pre- and post-session means and standard errors, their differences, and test statistics and p-values from the paired t-test. These data provide insights related to Kirkpatrick Level 2 evaluation, demonstrating that panelists did experience changes in managing stress and increasing mindfulness, resiliency, and empathy.

**Fig. 3:**
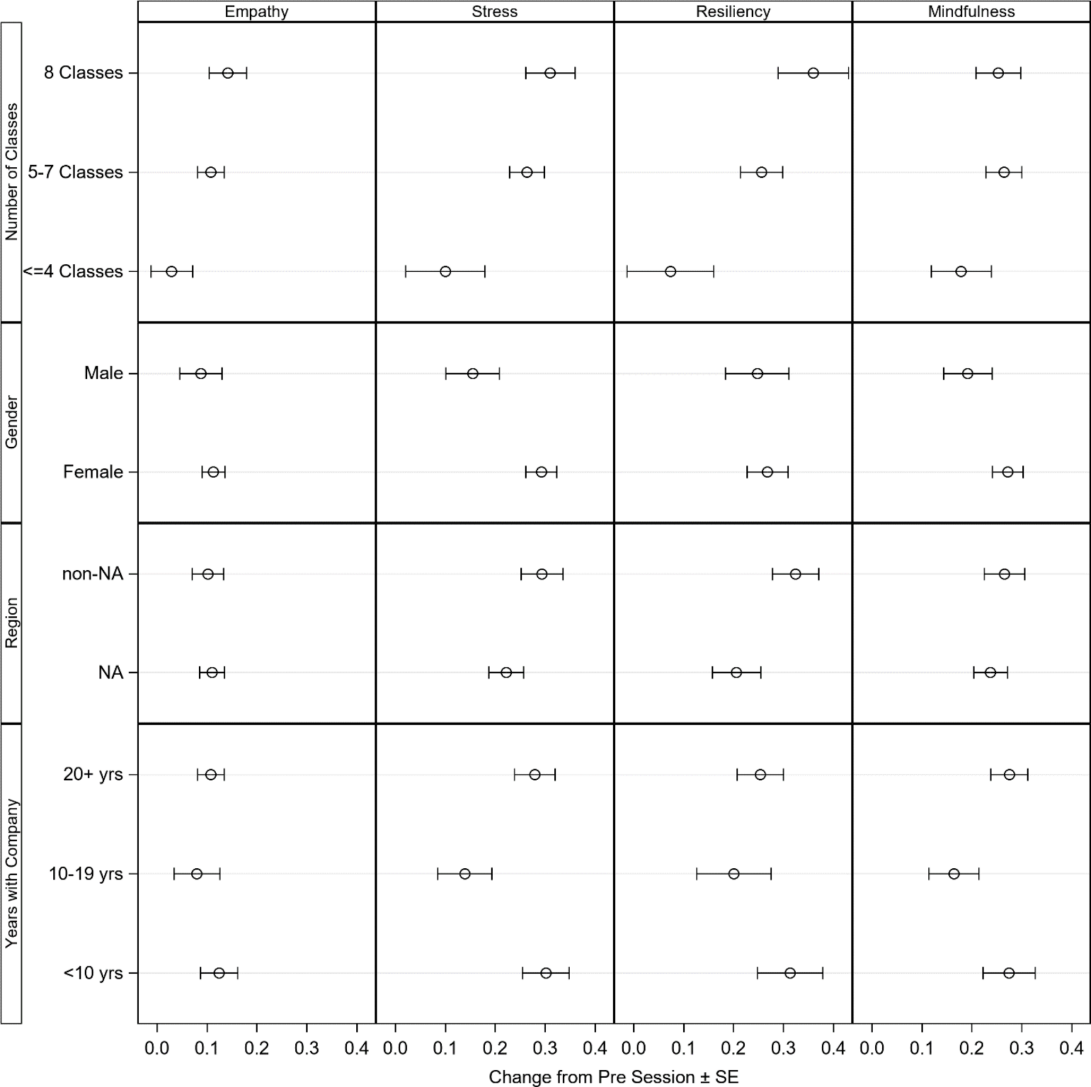
*Means and standard error bars demonstrate improvements for the four domains for each MWE participant subgroup, all sessions (N = 268). The x-axis shows change on the scale of 0 to 0.4 relative to per-session score; 0.0 occurs when there is no change*

The test subjects had significant improvements in all four domains, whereas the comparison group did not. Significant increases in stress management (*t* = 9.81; *P* < 0.0001), mindfulness (*t* = 9.18; *P* < 0.0001), empathy (*t* = 5.44; *P* < 0.0001), and resiliency (*t* = 7.93; *P* < 0.0001) are found regardless of gender, region of employment, tenure with the company, and whether the participant already had a regular meditation practice. Due to smaller sample sizes for employees working in some countries, and the hypothesis that subject familiarity with the English language might influence program effectiveness, region of employment is assessed in two groups: one for the US and Canada labelled as North America (NA) vs. all other countries (non-NA). Figure 3 shows the domain means and standard error for all subgroups, reflecting clear improvements across the board relative to when the mean is zero, which occurs if a subject’s pre-session score was identical to his/her/their post-session score (‘No change’).

Although most means and confidence intervals overlap across subgroups within a domain, there is a noticeable difference in the stress domain between women and men *(p* = 0.0263). Stress pre-course means are not different by gender: 2.95 for women and 2.98 for men, and none of the other domains show a significant difference between the two genders. One hypothesis for this difference is that men attended fewer classes on average, which is indeed the case from self-reported class attendance (20% of men attended 4 or fewer classes as compared to 11% of women).

**Fig. 4:**
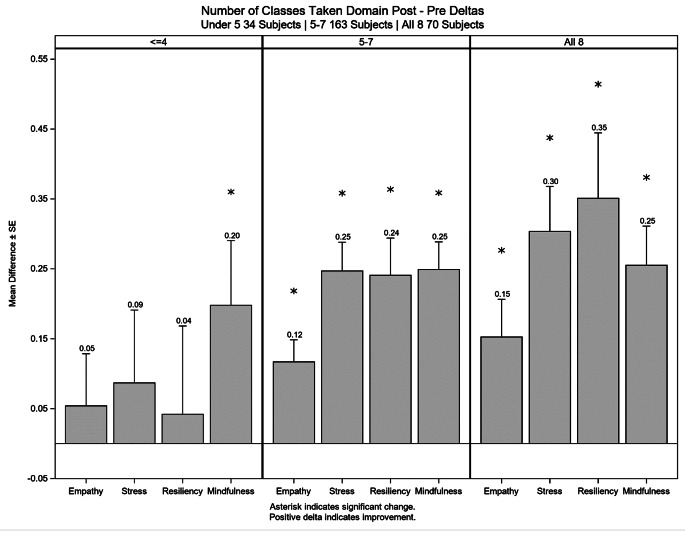
*Means and standard errors for each domain by number of classes taken. Asterisks indicate significant change*

Figure 4 displays the change means (standard error) by the number of classes taken. Subjects completing 4 or fewer classes have lower mean changes in every domain, which is the comparison group. The test group subjects completing more than 4 classes see significant improvement in every domain, whereas the group attending 4 or fewer classes only see significant change in the mindfulness domain.

### Post-program text analysis

An open-ended, voluntary comment question is included in the post-session surveys: “Please provide any additional comments, specific or general feedback on the course and your experiences.” These responses give information to evaluate the study at Kirkpatrick levels 1, 3, 4. Using the JMP text analysis platform, the second Word cloud in Fig. 1 is created from the open-ended comments, based on frequency of individual words or phrases. This Word cloud reveals general take-aways about what participants felt the MWE program delivers, such that participants learned and practiced many techniques, which they feel helped, and they would recommend the great course to others.

The MMC Analysis evaluates whole comments, rather than individual words or phrases. This AI-based model classified the verbatims, identifying that the intervention was successful according to most comments: these were rated as Good (13%), Great (65%), WOW (13%) and MMC (4%). Negative and Neutral were < 5% of comments received, and most were related to an inconvenient class time. 10% of responses included comments that the subject would continue his/her practice, implying habit formation.

Table 3 lists representative quotes for each MMC Analysis level. WOW comments are described as ‘best ever’ experiences with no behavior change, while MMC comments are described as a best experience with behavioral change and potential for new habit creation – which would fulfill an objective of the MWE program to support participants’ self-sufficiency to implement a long-term personal meditation practice. The WOW and MMC comments offer a unique perspective on which topics create the MWE participants’ delight. These statements can be used to inspire and help polish language used to market the program and help bring the program’s benefits to life (e.g., “This was a life changing course”, “I can apply the techniques when stress gets to me”, “I can focus better”, “I see positive outcomes in my professional and personal life”).

**Table 3:** *Representative quotes from anonymous MWE participants (N = 471) displaying the mental model change (MMC) classifications used in consumer products research and applied in this study to evaluate impact of the program*

Class	Representative verbatims for each Mental Model Change classification
**MMC**	- *This was a life changing course. I am not sure why we weren’t taught this in school, it is primal living 101 stuff. I went from not looking forward to the weekly class because it ‘interfered’ with work, to looking forward to the class and feeling more ‘in control’ at work, even though there isn’t less work. It was like a mid-day nap for the body AND soul. I will be joining all future sessions and making meditation a daily habit! Thank you!* - *This is the best course ever that I did in my career inside P&G particularly in this difficult time facing COVID situation. I keep saying that this was a gift that I received from Annie and I am grateful for that. I hope that everybody inside the Company could have the opportunity to receive it. Many thanks to really change my life. I keep listening that we as (Products Research) want to change our consumers’ life with new experiences, and now I was touched and had my life changed by this tremendous course. Keep doing that for P&G employees as most of them need it and never realize it. thanks.*
**WOW**	- *Best course and time investment! This course is about us as human being not human doing. The lessons I learned here helped a lot in my daily interactions with people. It helped a lot in my work, which means it helps P&G too. BIG THANK you Annie, Mark and whole team. I’ll continue to do the practice.* - *Deep knowledge and very structured and engaged way it was delivered. Overall, I loved it and I am seeing positive impact of it on my personal and professional life. I’ll continue with the sessions. I started without expectations, but even if I started with expectations, it would have exceeded over and beyond! Thank you.* - *It was a great learning and I can apply this when stress gets to me. It’s really effective after the meditation. I can focus more and I learn to pay more attention to details around me, people, environment, and the 5 senses really get to me, making me feel so confident as I focus. Helpful and each session I had I shared with my friends…* ;-) *Thanks for such great session. Would love to attend more.*
**Great**	- *A great course that brought my meditation skills forward. I strongly believe that meditation is key to better stress resilience and a healthier life. Big thanks to Annie for the inspiring, broadening journey.* - *Amazing. I wish we had a little more time together. Forgiveness of a person who was difficult had a profound effect on me. Thank you so much!* - *The course has given perspectives to learn how to control oneself and keep focus. The techniques were wonderful and by doing this course, I am able to manage stress in a better way. Thanks a lot for these sessions. Hope to attend future sessions.* - *I would like to take this course again just to reinforce the teachings. Thank you for providing the presentations for reference, and for providing these classes.*
**Good**	- *(MWE course) was very good and taught many techniques. Probably I would need more self-practice to get even bigger value out of it.* - *The course opened my mind to new and different ways to meditate. I had a narrow view of what meditation was and how to do it. This course also helped me to realize meditation has different purposes, which are not all relaxing and calming, but uncomfortable and emotional at times. Though some of the practices did not fully resonate with me, I believe they were still important to learn and practice as there may/will come a time I may need that type of meditation to get unstuck in some situations.​*
**Neutral**	- *Is it possible to offer this in US afternoon next time to avoid Europe and Asia meetings?* - *I had strong interest in this course but was not really a good student. I was not concentrated due to the timing (midnight). Hope I have a chance to get this (on) a bit earlier timing.*
**Negative**	- *I may not be in the right space yet, but this meditation did not help me. I got distracted and bored easily. I’m open to taking it again sometime in the future, maybe the time will be right for me then.* - *Unfortunately, I had to miss the conversations at the end because I had another meeting at 12:15. I do wish had more time to go ‘deeper’.*

## Discussion

### Implications for theory and practice

As anticipated from the SIOP top trends in occupational health, the Gallop emotional experiences poll of people in > 100 countries, and other large surveys, the MWE enrollee demographics show that interest in this kind of intervention is global and spans across all adult generations and genders (Table 1). Having thousands of employees enroll over multiple sessions is also a good indicator of the widespread interest in the advertised objectives of the program and its perceived effectiveness inside the organization.

The first Word cloud and Topics, gleaned by text analysis of the enrollees’ objectives, highlight the value proposition of the program, which can be used to inform marketing messages about the intervention (Fig. 1). There are distinct and overlapping needs expressed for what enrollees want to learn and practice to improve their work performance and personal lives. The combination of these objectives is not surprising given that, logically speaking, one would attain conditions of reduced stress and distraction by learning skills to (re)train the mind to respond to situations differently, improving mental and emotional wellbeing.

Test statistics and p-values from this research indicate the MWE program meaningfully delivered on the learning objectives or needs expressed by the employees (Table 2). The pre-program mean for the empathy domain was high (3.77 of 5), and despite the range restriction, subjects still showed a significant increase (to 3.88) – addressing the common objective to improve emotional intelligence aspects. The significant increase in subjects’ reported mindfulness is relevant for the common objective to increase focus and reduce distraction. The significant reductions in the overloaded and uncontrolled subdomains directly match employees’ desire to feel less stressed, to be at peace and feel calm. Subjects’ resilience was also significantly increased, which is related to all their learning objectives, notably learning new techniques to improve mental and emotional perspectives and responses to difficult situations. Subjects saw these benefits regardless of gender, region of employment (estimate of nationality), and length of time employed by the company (estimate of age). There is also consistency in the domain mean slopes across the 3 sessions in this study, showing that people are in similar starting places and have similar growth, suggesting the positive outcome is not a fluke (Fig. 3). The only group which did not see significant benefits across all four domains are those in the comparison group. Although they still showed some improvement in mindfulness, the overall result raises the question of whether a shorter series of this or any meditation-related program could result in subject improvement in these four important domains.

Most participants that dropped out of the MWE program, attending 4 or fewer classes, cited other work priorities or time conflicts and were not prepared to modify their schedules to accommodate attending the 50-min class once a week. This action implies they were ambivalent about taking consistent action to learn and practice the skills, indicating they may have been in a TTM Contemplation stage of behavior change at the onset of the program. Although attending fewer than 4 of the 8 classes results in less learning and no significant change in 3 of 4 domains, that approach would support their collecting information, thinking about the concepts and practices, and in many cases inspiring them to fully attend a later session. Hundreds of people repeatedly take the MWE course, theoretically moving themselves from TTM Contemplation stage to TTM Action stage over months or years. People in the TTM Precontemplation stage are characterized as resistant to making changes in their relevant habits/behaviors, and therefore it is assumed they would not voluntarily enroll in the program.

This is a notable finding for organizations introducing wellbeing concepts through single workshops or practices; such short events may provide helpful information but should not be expected to affect behavior or reduce stress or distraction, for example.

The hypothesis that less familiarity with English, the language of class instruction, could be a barrier to improvement appears to be unfounded for the population in this study. Employees working in all regions demonstrate significant improvements in all domains (Fig. 3). It is unknown why employees working in more than 30 countries outside North America, and thus assumed to not be native or fluent in English, had higher mean changes in 3 of 4 domains than employees working in North America, presumed to be native English speakers. Results imply these corporate employees are sufficiently proficient in English to learn the meditation techniques and practice them effectively. There may be a cultural difference in learning diverse resilience skills, based on the apparent large difference in the resilience domain for employees working in North America (lower) vs. outside North America (higher); this difference could not be attributed to the number of classes attended, gender, or years of employment.

The difference in the mean changes in the stress domain between women and men appears to be due to men attending fewer classes. There are also known differences in how men and women react to and report physical and mental stress, and different coping strategies (APA, 2010).

Figure 3 also appears to show a moderate difference in mean changes in stress and mindfulness in the employee subgroup for duration of employment, which is loosely associated with age or generation at this company. Although all the domain changes are significant, employees in mid-career (10–19 years of employment) appear to have less mean change than the young new hires and employees with more than 20 years of employment at this company. This figure demonstrates the importance of including confidence intervals in evaluations; accounting for both means and standard error, there is no statistically significant difference between the subgroups for duration of employment. Although a U-shaped happiness-age curve is actively debated, people in this mid-age range/life stage may be less happy than the other age groups. A recent report examines data from half a million Americans and West Europeans and concludes both males and females reach their lowest happiness point in middle age (Blanchflower, 2021). Most participants in this MWE study are from the US and Western Europe.

Findings from all the qualitative data, comments collected before and after session participation, appear to confirm that the MWE intervention is successful. Analysis of subject objectives prior to attending the sessions show that the main goals were taking time to practice and learn, improving mental health, reducing stress, and improving balance, focus, and concentration. After the sessions, subjects reported stress reduction, improved focus, appreciation that the company offered the sessions, improved work life balance and that they would recommend the course for coworkers. Classification of the comments using a mental model change analysis showed 83% of the comments were ‘great’ or higher.

The word cloud (Fig. 1) and MMC classification results of post-session open-ended comments (Table 3) indicate people felt the MWE intervention aligns with what they hoped to learn and is a good value for their time investment. By covering 8 techniques, people felt empowered to learn and select the meditation type(s) that work best for them in their current situation. Company sponsorship of the 2 months of dedicated practice time, especially for those who could join class during their workday, demonstrated that wellbeing is indeed a company priority and that learning/practicing the skills was acceptable if not encouraged. People appreciated that they could apply what they learned both inside and outside work. Finally, many said they recommended MWE as a great program, and would encourage their colleagues to take it because it helped them.

The insight gained from the open-ended comments, and the emotions expressed therein, would be missed in a study that only evaluates intervention success using quantitative standard questionnaires. The detailed analysis of comments ‘by hand’ of consumer research and wellbeing experts reading over the thousand texts, in combination with the MMC classifications, enable those marketing the program to extract inspiring quotes as well as identify concepts or trends that could motivate people to take advantage of the program and participate.

Productivity is a priority especially within large industries, so interventions that significantly improve employee health aspects that impact job performance can be viewed as a high return on investment. The MWE program results imply that for a relatively short time investment - people learning and practicing for an hour or two per week for a couple months - employees can feel better and be more productive due to less stress, and more resilience and mindfulness. The increase in empathy may also support company diversity and inclusion goals that encourage individuals to feel safe to be themselves at work and be respected.

### Lessons learned: characteristics for an effective mental and emotional wellbeing intervention

Table 4 highlights characteristics that are attributed to making the MWE program effective for mental and emotional wellbeing of employees at this global corporation. Key aspects for a successful intervention all revolve around encouraging and enabling consistent employee participation. Characteristics include:

- Very specific to address what employees know they want or need to learn;
- Format incorporates core adult learning principles and health coaching strategies;
- Exposes participants to practices that increase awareness, discipline, compassion, and insight;
- Targets the most relevant TTM behavior stage to meet the needs;
- Led by a trusted, very experienced and thus credible teacher/coach;
- Company sponsored and endorsed;
- Builds community;
- Evaluate the intervention in a way that addresses the 4 levels of the Kirkpatrick model.

These attributes are important given that quite a few MBP programs are not proven effective as intended (Eby et al.).

**Table 4:** *Characteristics that make the MWE program effective as a mental and emotional wellbeing intervention*

Characteristics	Description of how to address the characteristic in an intervention
Very specific to address what employees know they want or need to learn	Determining what adults want or need to learn involves a preparatory survey with open-ended questions so people can elaborate on their situation. In this study, employees listed their objectives when registering for the course, which were to: (1) Reduce anxiety, distraction, negativity about self and world, (2) Increase adoption of essential mental and emotional wellbeing skills, (3) Improve quality of life and work performance (e.g., increased focus, patience). Although the objectives are clear, more insight into people’s situations and skills desired is needed. For example, an employee may desire ‘stress reduction’ but the specific skills they need are unknown or vague to them. ‘Stress reduction’ is too general for a program to target, so distinguishing specific and sometimes overlapping causes for the stress is important. Continuing the example, further questions identify that an employee’s workload doubled over the past 3 years, and the person feels they cannot decline the new work nor complete the work adequately, which sets up a ‘no-win situation’ upon which they ruminate and feel stressed. Thus, an emotional/mental wellbeing intervention might target (a) bringing awareness to the emotions like fear of talking with a supervisor to reduce workload, fear of being viewed as inadequate by a superior or peers, disappointment in their own work performance, derogatory inner speech, and/or (b) practicing skills on nonviolent communication or crucial conversations, or how to reflect on the value of their work and personal health in a structured way.
Format incorporates core adult learning principles and health coaching strategies	Actively integrating key learning principles in course format improves the likelihood that employees will realize the benefits of meditation and become inspired and self-sufficient in maintaining their own meditation practice. It begins with course announcements, which communicate common learning objectives and offer it several times during a year and in different time zones to permit employees to participate when they realize they want/need such training. The background information and selected practices shared during a session respond directly to what people realize they want/need to learn now, enable self-efficacy through repeated practices, and build autonomy to explore techniques and self-direct. Each class format is consistent with the CRAFT model used in coaching: Check-in to build rapport across the community and with the teacher, participants Report their progress, Adjust/Assign techniques to practice, teacher collects Feedback and Teach/train concepts.
Exposes participants to practices that increase awareness, discipline, compassion, and insight	The 8 meditation techniques in the MWE program are taught in a specific order to build on the previous practices. The first meditations are to increase Awareness (sensing), later techniques help people manage distractions and practice Discipline (focused attention), open more to Compassion (intent toward self and others), and gain Insight (reflection) into who we really are, and how we might live and work with more joy, less effort or strain.
Targets the most relevant TTM behavior stage to meet the needs	The common general needs to reduce stress, improve focus, and improve emotional intelligence represent a complicated set of behaviors and psychological perspectives, not adequately addressed by reading a couple articles or attending a 3-hour workshop. To support people to change their behaviors and mindsets to be able to take consistent and effective new actions and maintain their practice of meditation skills over months or years, this program specifically targets people who were already preparing for change and ready to take action (TTM Preparation and Action stages of change). Since these behavior stages take months to fulfill, a longer program, such as one meeting regularly over 8-weeks with options to continue throughout the year, is needed.
Is led by a trusted, caring, and experienced teacher/coach	A client/employee’s trust in the coach/teacher is essential for a well-functioning interaction. The learning process progresses more quickly and is more effective when there is strong rapport. Studies in the Journal of Trust Research indicate that when a coach communicates more benevolence, clients feel more autonomous, one of the core adult learning principles.
Company sponsored and endorsed	Employee commitment to participation is reassured by having a company-supported program offered during the workday. Official company sponsorship aids in reducing potential negative stigma associated with a person acknowledging simply by attending a group session that they might not have ideal mental and emotional health, or related performance or work-life balance concerns. When leaders encourage their team members to participate, and they role model techniques that improve personal wellbeing, teams are inspired to learn and practice as well.
Builds community	Having a sense of community strengthens accountability, in part because joining a group session implies a public commitment to learn and practice. The MWE program encourages participants to respect privacy, and then feel safe to text or verbalize their experiences and questions during class, enabling others to hear different experiences with a meditation technique. Post-session feedback shows that participants appreciated the weekly feedback/reporting discussions and enjoyed being part of a regular practice community for encouragement, accountability, insight, and friendship. The sense of community also encourages people to meet and continue to practice after the sessions, supporting people to progress from the TTM Action to Maintenance stage of behavior change. Seeing faces and names from all over the world also inspired participants to feel like one family, working together, less alone with their achievements and difficult situations.
Evaluate the intervention in a way that addresses the 4 levels of the Kirkpatrick model	The Kirkpatrick evaluation model is purported to be a global standard for demonstrating effectiveness of training programs. Using both quantitative and qualitative feedback on an intervention, including both standard closed- and open-ended questions, organizers can assess participants’ reaction, learning, behavior, and results. Levels 1–4 assesses how favorable and relevant the training is to the learner, whether and how well participants acquired the skills, if/how participants applied what they learned in work or personal settings, and if targeted outcomes (like stress reduction) were achieved. Evaluation is critical to ensuring return on investment, that a program delivers desired results.

### Limitations and recommendations for future research

As with many studies with volunteers, researchers cannot force people to respond to surveys and thus might lack insight into why people do not complete the course or not respond to the survey request. Survey links were sent to all enrollees (~ 3300); 60% of those completed a pre-session survey and 20% completed a post-session survey. Individual attendance could not be tracked but based on attendance numbers, it appears about 30% of enrollees might not have called in to any class, and about 35% of the original enrollees attend most of the classes in a session. Although this implies a higher rate completion of the pre- and post- session surveys by genuine participants, this also implies a significant number of people enrolled but did not attend or did not fill in a survey for unknown reasons.

This research shows that outcomes of an effective intervention can be measured with a validated pre-post session survey and text analytics of unstructured text. Since some comments in the post-session survey allude to participants continuing their meditation practices after completing the course, it is recommended that surveys of these subjects be conducted at 6- or 12-month intervals to determine if or how long the positive changes persist. A future study would include questions to an active control group that does not attend any class in the program, questions from all 3 subdomains from the stress standard survey, and an analysis of the reliability of each standard question for this global corporate population to identify the most reliable questions and abbreviate the long list of questions to only ask those. Since the consumer products qualitative research methods yielded valuable insight, we recommend applying the combined approach of using both the quantitative pre-post standard surveys and the qualitative analysis of participants’ comments to other wellbeing interventions offered at the company.

## Conclusion

This study indicates that the MWE course is very positively received and supports corporate employees around the world in significantly improving their stress, mindfulness, resiliency, and empathy by providing them with the tools and practice they need to feel focused, capable, in control, and supported by their colleagues and organization. The study also shows that reapplying methods from advanced consumer products research is effective for determining the value proposition that will help promote engagement among employees and investigating the impact of employee wellbeing interventions. Established standardized surveys of stress, mindfulness, resilience and empathy yield quantitative pre-post data to evaluate a program, fulfilling Level 2 of the Kirkpatrick evaluation model related to participant Learning. Studies that use novel methods like text analytics and reliable NLP models can deliver a more comprehensive evaluation of Level 1 - participant enjoyment and relevance of the program to meet their needs, Level 3 - behavioral change resulting from training/coaching experience, and Level 4 - impact on a person’s health and work performance. Both traditional surveys and cutting-edge AI models that reveal trends from open comments can guide organizations toward impactful programs that truly support their employees’ wellbeing interests and needs.

## Data Availability

The datasets generated during and/or analyzed during the study are available from the corresponding author on reasonable request.
